# Rich Organic Nitrogen Impacts Clavulanic Acid Biosynthesis through the Arginine Metabolic Pathway in Streptomyces clavuligerus F613-1

**DOI:** 10.1128/spectrum.02017-22

**Published:** 2022-12-14

**Authors:** Jiafang Fu, Xinru Xie, Shaowei Zhang, Ni Kang, Gongli Zong, Peipei Zhang, Guangxiang Cao

**Affiliations:** a Biomedical Sciences College, Shandong First Medical University, Jinan, China; b NHC Key Laboratory of Biotechnology Drugs, Shandong Academy of Medical Sciences, Jinan, China; Forschungszentrum Jülich GmbH

**Keywords:** clavulanic acid, arginine metabolism, metabolome, transcriptome

## Abstract

Clavulanic acid (CA) is the preferred clinical drug for the treatment of infections by β-lactam antibiotic-resistant bacteria. CA is produced by Streptomyces clavuligerus, and although there have been many reports on the effects of carbon and nitrogen sources on CA production, the mechanisms involved remain unclear. In this study, we found that CA accumulation in *S. clavuligerus* F613-1 was increased significantly in MH medium, which is rich in organic nitrogen, compared with that in ML medium, which contains half the amount of organic nitrogen present in MH medium. Transcriptome analysis revealed that genes involved in CA biosynthesis, such as *ceas1*, *ceas2*, *bls1*, *bls2*, *cas2*, *pah2*, *gcaS*, and *cad*, and arginine biosynthesis, such as *argB*, *argC*, *argD*, *argG*, *argH*, *argJ*, and *argR*, were upregulated under rich organic nitrogen. Metabolome data revealed notable differences between cultures of F613-1 grown in MH and ML media with regard to levels of key intracellular metabolites, most of which are involved in arginine metabolic pathways, including arginine, glutamine, and glutamic acid. Additionally, supplementation of ML medium with arginine, glutamine, or glutamic acid resulted in increased CA production by *S. clavuligerus* F613-1. Our results indicate that rich organic nitrogen mainly affects CA biosynthesis by increasing the levels of amino acids associated with the arginine metabolic pathway and activating the expression of the CA biosynthetic gene cluster. These findings provide important insights for improving medium optimization and engineering of *S. clavuligerus* F613-1 for high-yield production of CA.

**IMPORTANCE** The bacterium Streptomyces clavuligerus is used for the industrial production of the broad-spectrum β-lactamase inhibitor clavulanic acid (CA). However, much remains unknown about the factors which affect CA yields. We investigated the effects of different levels of organic nitrogen on CA production. Our analyses indicate that higher organic nitrogen levels were associated with increased CA yields and increased levels of arginine biosynthesis. Further analyses supported the relationship between arginine metabolism and CA production and demonstrated that increasing the levels of arginine or associated amino acids could boost CA yields. These findings suggest approaches for improving the production of this clinically important antibiotic.

## INTRODUCTION

Many antibiotic-resistant bacterial strains have emerged due to the long-term, widespread use of β-lactam antibiotics. The main antibiotic resistance mechanism of these strains is the production of β-lactamase, which degrades and inactivates β-lactam antibiotics. As a broad-spectrum β-lactamase inhibitor (1–3), clavulanic acid (CA) is often used in combination with penicillin or cephalosporin derivatives, an approach which can effectively improve the antibacterial effect of β-lactam antibiotics and has been widely used in the clinical treatment of infections by β-lactam antibiotic-resistant bacteria (4–6).

CA is mainly produced by Streptomyces clavuligerus (7) through a complex biosynthetic pathway that involves two direct precursors, arginine and glyceraldehyde 3-phosphate (G3P) (8, 9). In the initial step of the pathway, arginine and G3P are synthesized into N^2^-(2-carboxyethyl)-arginine (CEA) by CEA synthetase, which is a key initiating enzyme for CA synthesis and is encoded by two homologous genes, *ceas1* or *ceas2* (10). CEA is then catalyzed by β-lactam synthetase (β-LS, encoded by the *bls1* or *bls2* gene) to form amide bonds within the molecule and produce deoxyguanidino-proclavaminic acid (DGPC) (11–13). DGPC is further catalyzed by clavaminate synthase (Cas, encoded by the *cas1* or *cas2* gene) to form guanidinoproclavaminic acid (14–16). Proclavaminicaminidinohydrolase (Pah, encoded by the *pah2* gene) catalyzes the removal of guanidine from guanidinoproclavaminic acid to form proclavaminic acid (17). Next, proclavaminic acid is converted to clavaminic acid by two steps of a continuous reaction catalyzed by Cas (18). Clavaminic acid is a branching point for CA and 5S clavams. In subsequent reactions, a portion of the accumulated clavaminic acid is converted to CA by several enzymes, encoded by *cad*, *cyp450,* and *oppA1*, and the remaining clavaminic acid is used to form a variety of 5S clavams through a series of enzymatic reactions (19–21).

Although the CA biosynthetic process has been largely elucidated, the mechanisms regulating the metabolic flux between primary metabolism (especially arginine and G3P metabolism) and CA biosynthesis in *S. clavuligerus* are not yet clear. Several reports have indicated that different sources and amounts of organic nitrogen affect CA production (9, 22, 23). However, there have been no in-depth reports on how the types and content of organic nitrogen sources affect CA biosynthesis. In this study, we investigated the effects of organic nitrogen on CA biosynthesis in the strain *S. clavuligerus* F613-1 through metabolome and transcriptome analyses, and our findings provide insights into medium optimization and fermentation design for improving CA yield.

## RESULTS

### Impact of different organic nitrogen concentrations on growth and CA production of strain F613-1.

**FIG 1:**
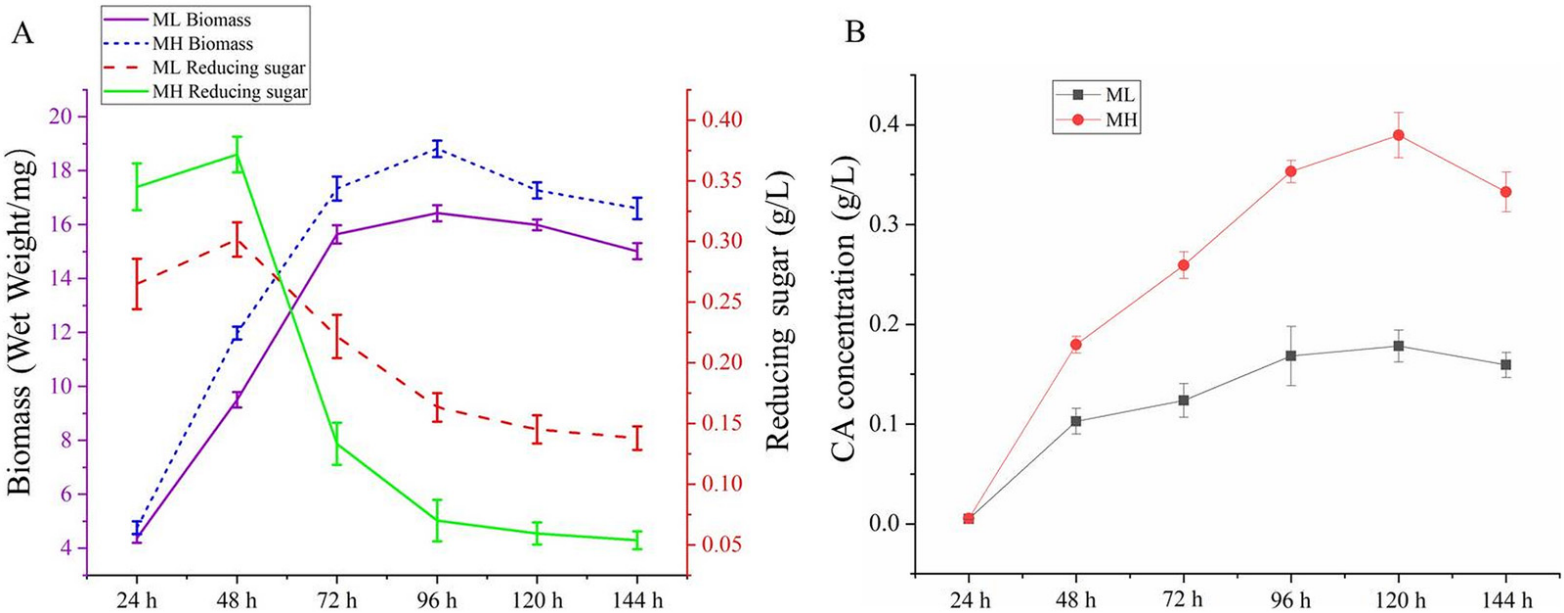
Growth and clavulanic acid (CA) production of F613-1. MH and ML refer to medium containing 0.8% peptone or 0.4% peptone from soybean powder, respectively. (A) Growth curves, determined by biomass and concentration of reducing sugar in MH or ML medium. (B) Analysis of the change in CA production during CA fermentation. Data are the means ± standard deviation (SD) of six separate experiments.

Several studies have reported that soybean powder or soy protein isolate affects CA production (9, 22, 23). We used 0.8% peptone (MH fermentation medium) and 0.4% peptone (ML fermentation medium) derived from soybean powder to investigate how organic nitrogen impacts growth, CA biosynthesis, and fermentation characteristics (Fig. 1). Results showed that the biomass of F613-1 increased exponentially over 72 h of cultivation, and then the cultures entered stationary phase (72 to 144 h) in both MH and ML media, with a slightly higher biomass in MH medium (Fig. 1A). The reducing sugar content in MH medium was higher than that in ML medium during the logarithmic growth prophase (Fig. 1A), coinciding with the early stage of CA biosynthesis (Fig. 1B), followed by a sharp reduction at 48 to 72 h and then a more gradual reduction to notably lower levels than in ML medium (Fig. 1A). Concomitantly, the CA accumulation was markedly higher in MH medium than in ML medium (Fig. 1B).

### Distinct metabolite profiles in MH and ML media associated with CA production.

**FIG 2:**
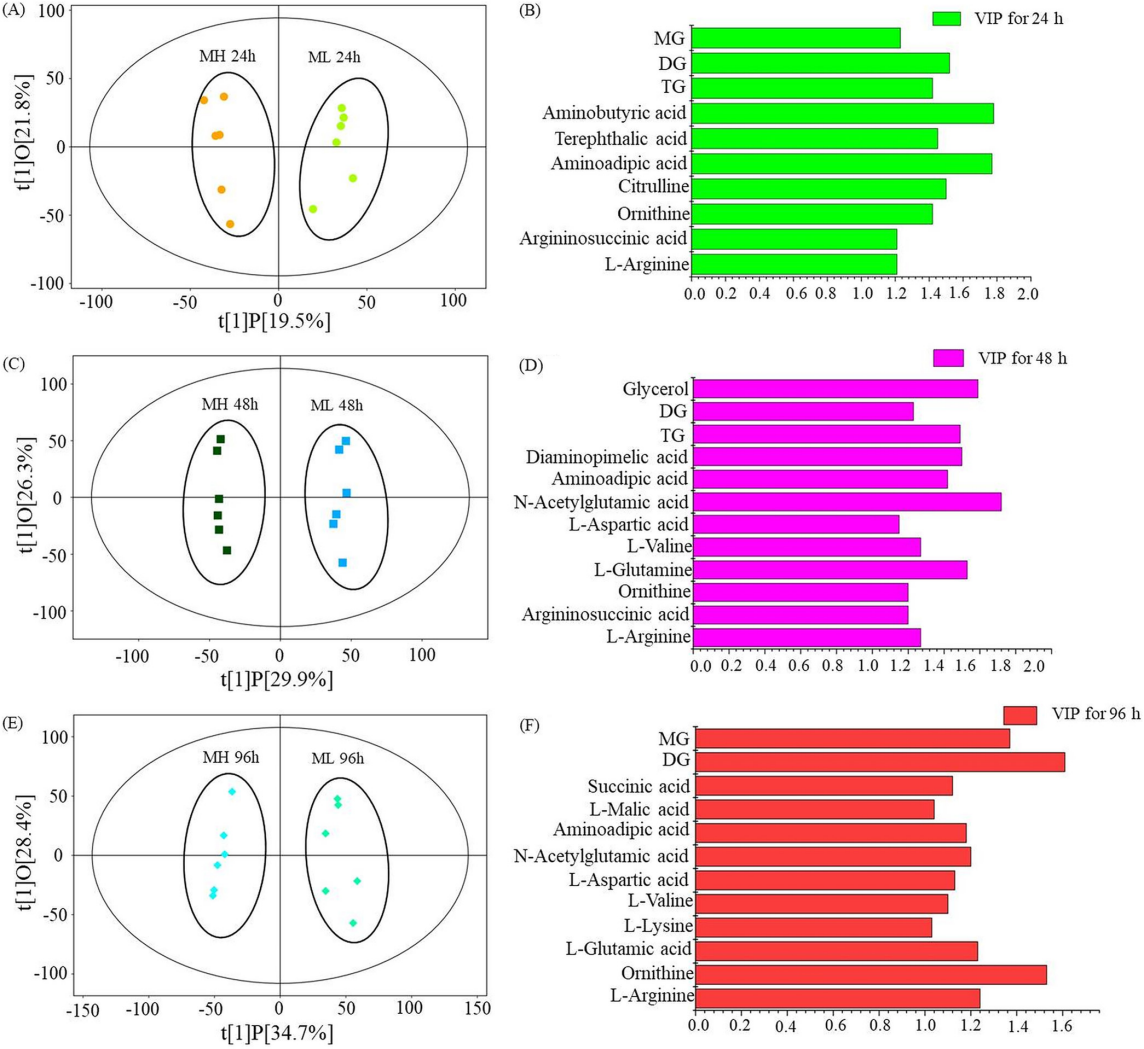
Orthogonal projections to latent structures-discriminant analysis (OPLS-DA) of intracellular metabolites in samples from MH and ML media. (A, C, and E) Score scatterplots of the OPLS-DA model for (A) MH versus ML at 24 h, (C) MH versus ML at 48 h and (E) MH versus ML at 72 h. (B, D, and F) Differentially expressed metabolites with a variable importance in projection (VIP) score of >1 at (B) 24 h, (D) 48 h, and (F) 96 h. TG, glycerol trioleate; DG, glycerol dioleate; MG, glycerol monooleate.

Intracellular metabolites, including water-soluble compounds and fat-soluble compounds such as amino acids, sugars, organic acids, fatty acids, and other compounds, were compared between MH medium and ML medium by metabolome and lipidome analysis. The intracellular metabolites of samples from MH medium and ML medium differed markedly, as shown in the orthogonal projections to latent structures-discriminant analysis (OPLS-DA) score scatterplot obtained using different time points, with 24, 48, and 96 h corresponding to the early, rapid, and later stages of CA biosynthesis, respectively (Fig. 2A, C, and E). The promoting/antagonistic relationship of intracellular metabolites in biological processes was represented by variable importance in the projection (VIP) scores, and metabolites with a VIP score of >1 were selected as the key metabolites closely related to CA production (Fig. 2B, D, and F). These key intracellular metabolites had noticeably different concentrations in MH and ML media (Table S1), and they were mainly involved in arginine metabolic pathways (l-arginine, argininosuccinic acid, citrulline, ornithine, *N*-acetylornithine, *N*-acetylglutamic acid, l-glutamic acid, and l-glutamine) and lipid metabolic pathways (glycerol trioleate, glycerol dioleate, and glycerol monooleate).

**FIG 3:**
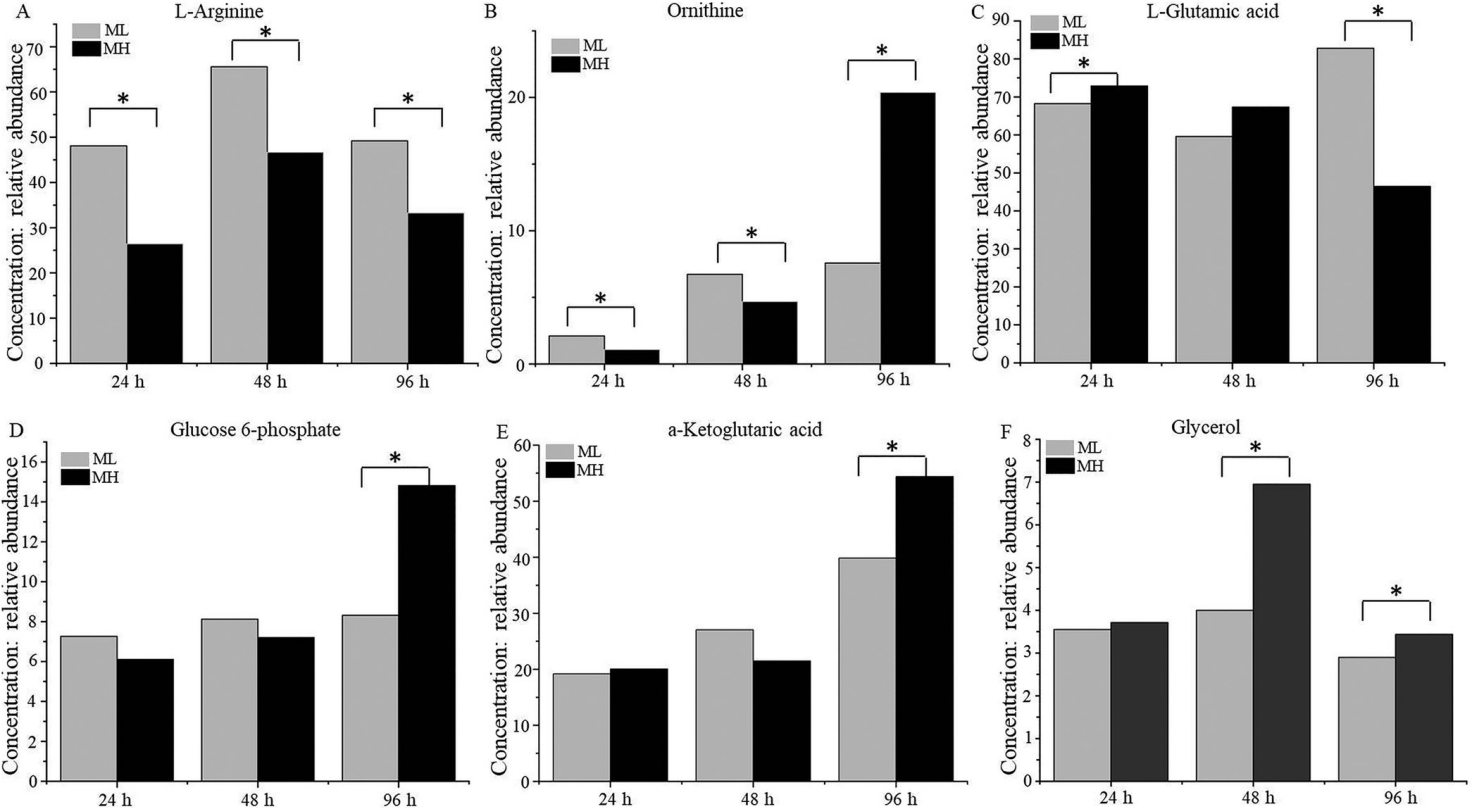
Comparison of the key metabolites closely related to CA production in Streptomyces clavuligerus F613-1 cultured in MH or ML media. Changes in concentration of l-arginine (A), ornithine (B), l-glutamic acid (C), glucose 6-phosphate (D), α-ketoglutaric acid (E), and glycerol (F) during CA fermentation. *, VIP score > 1.

Arginine is a direct precursor of CA biosynthesis (24). Although MH medium has twice the amount of organic nitrogen compared to ML medium, the intracellular l-arginine content of F613-1 decreased by 45.06%, 28.88%, and 32.37% at 24, 48, and 96 h, respectively, in MH medium compared with levels in ML medium during fermentation (Fig. 3A), indicating a more robust utilization of arginine in MH medium. Ornithine is the precursor of l-arginine, and the intracellular ornithine content of F613-1 decreased by 49.05% and 30.41% at 24 and 48 h (the early and rapid stages of CA biosynthesis), respectively, in MH medium compared with that in ML medium, but increased by 1.69-fold at 96 h (Fig. 3B). In addition, *argJ* (responsible for converting *N*-acetylornithine into ornithine) was significantly upregulated at 96 h in the MH medium compared with that in the ML medium (Table S5). The intracellular l-glutamic acid content increased slightly by 6.78% and 12.92% at 24 and 48 h, respectively, in MH medium but markedly decreased by 43.93% at 96 h in this medium compared with levels in ML medium (Fig. 3C), consistent with the impact of organic nitrogen levels on amino acids associated with arginine biosynthesis.

In contrast, most of the carbohydrate content, including glucose 6-phosphate and α-ketoglutaric acid, was similar in MH and ML media, although levels were higher (increased by 78.22% and 36.53%, respectively) in MH medium at 96 h (Fig. 3D and E). Additionally, the intracellular glycerol content of F613-1 increased by 73.93% at 48 h and 18.28% at 96 h in MH medium compared to that in ML medium (Fig. 3F). These results indicate that organic nitrogen content mainly affects CA biosynthesis by impacting the metabolic pathway of the direct precursor arginine.

### Effects of rich organic nitrogen on CA biosynthesis and transcription of associated genes.

To further explore how rich organic nitrogen affects CA biosynthesis, we performed transcriptome analysis. Three gene clusters are associated with CA biosynthesis in *S. clavuligerus*: the CA biosynthetic gene cluster, the paralog gene cluster, and the clavam gene cluster (25–28). Transcriptome data revealed that the transcriptional levels of these three gene clusters were high at 24 h, then decreased slightly at 48 h, and were significantly decreased at 96 h in MH medium (Fig. S1 and Table S2). In ML medium, these three gene clusters were also expressed most highly at 24 h, but with greater decreases in expression at 48 h (Fig. S1 and Table S3). In addition, most of these genes were slightly downregulated at 48 and 24 h in MH medium compared with levels in ML medium (Tables S2 and S3).

**FIG 4:**
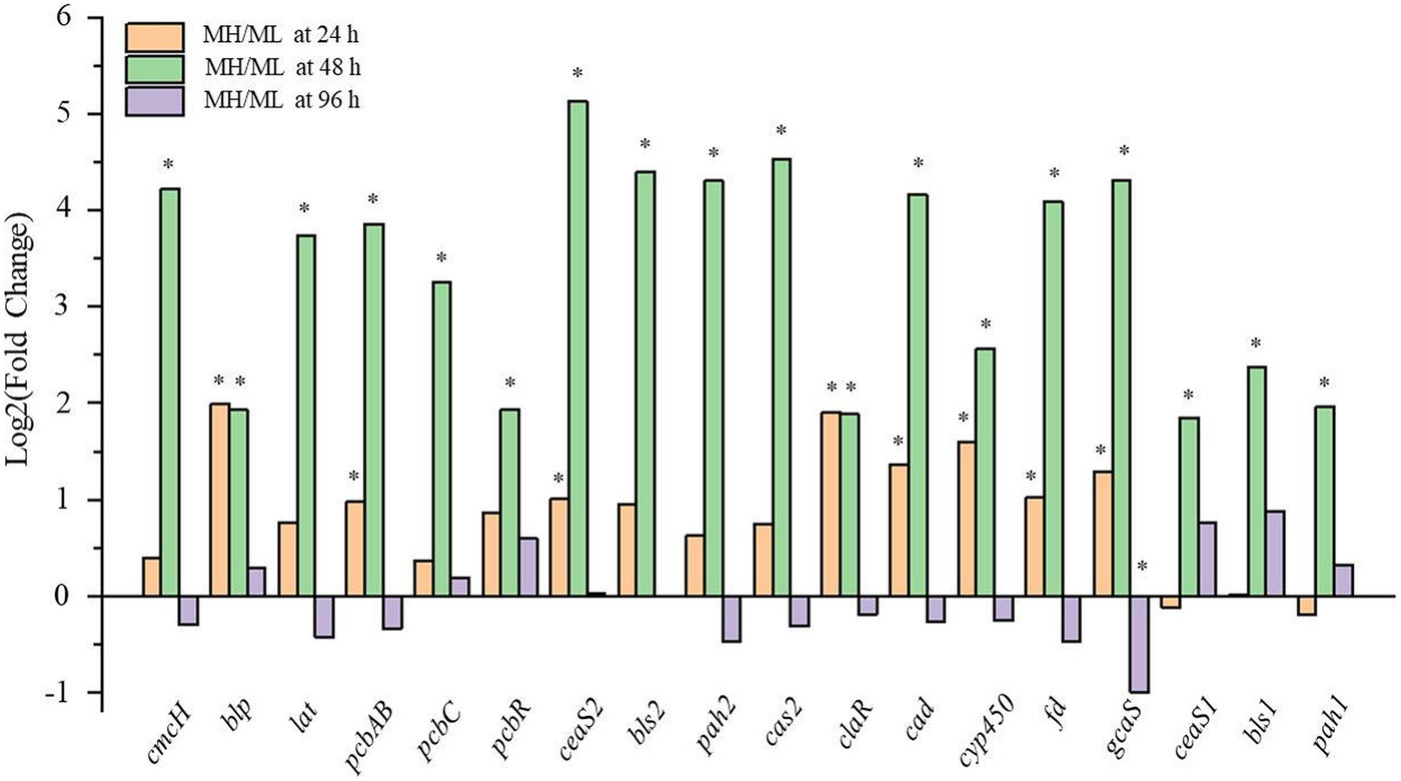
Comparison of the transcriptional levels of key genes in CA and cephalomycin C biosynthesis in MH and ML media. Results show log_2_ fold change in MH medium compared with levels in ML medium at 24, 48, and 96 h. *, *P* < 0.05.

Compared with the levels in ML medium, the transcriptional levels of these three gene clusters increased by 2- to 4-fold at 24 h and 2- to 32-fold at 48 h in MH medium, but had begun to decrease by 96 h, resulting in no significant differences in expression for most genes in the two media by the final time point (Table S4). Real-time quantitative PCR (RT-qPCR) data revealed that the expression trends of *ceas1*, *ceas2*, *bls1*, *bls2*, *cas2*, *pah2*, *pah1*, *gcaS*, *fd*, *cyp450*, *claR*, and *cad* were similar to the expression trend obtained from the transcriptome results (Fig. S2). The transcriptional levels of key genes involved in CA biosynthesis were slightly higher at 24 h, significantly higher at 48 h, and then similar at 96 h in MH medium compared with levels in ML medium (Fig. 4). These results indicate that rich organic nitrogen promotes CA biosynthesis by impacting the expression of the three CA gene clusters.

### Effects of rich organic nitrogen on arginine metabolism-related gene transcription.

**FIG 5:**
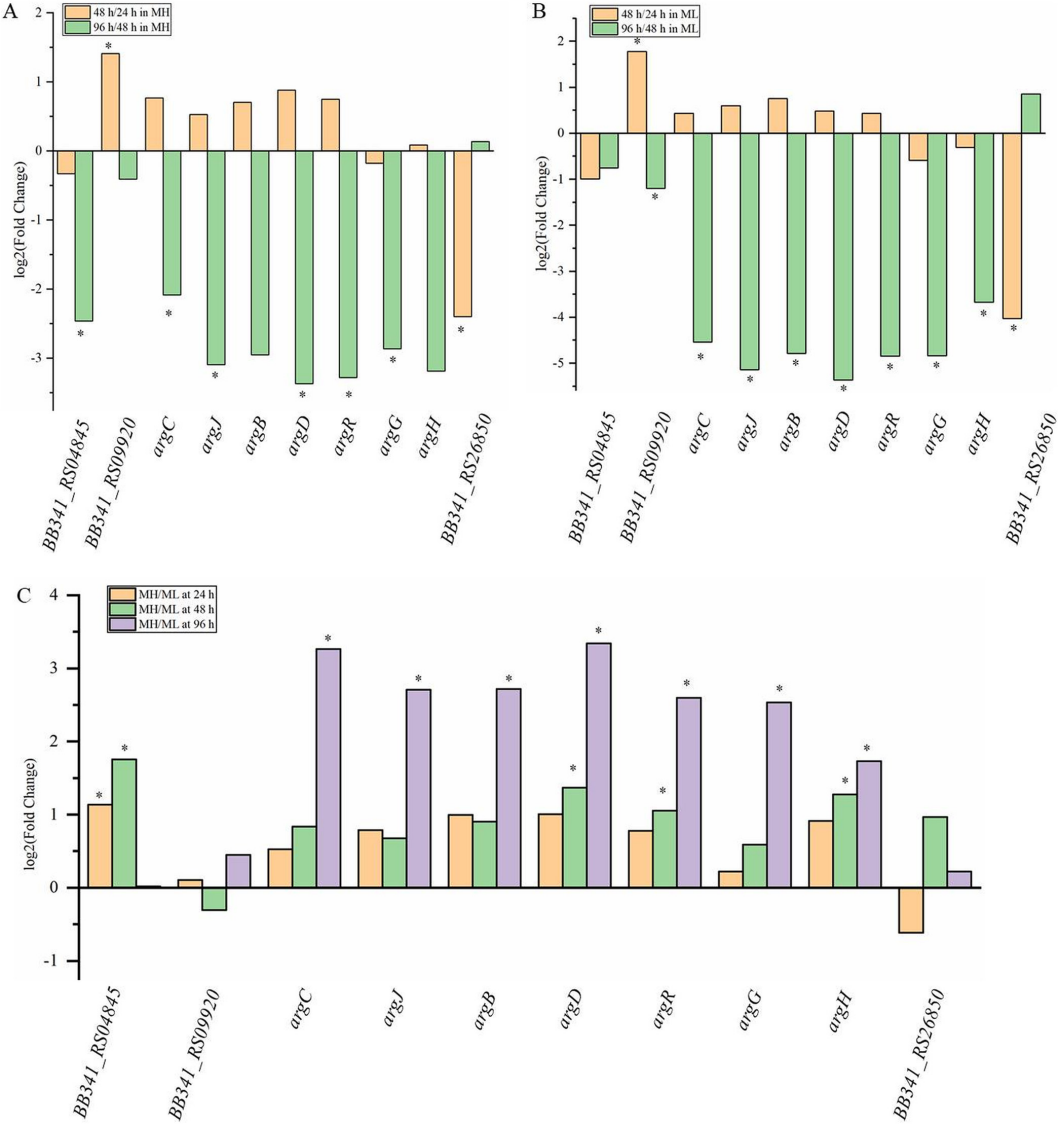
Comparison of the transcriptional levels of genes involved in arginine metabolism in MH and ML media. Results show log_2_ fold change at different time points in MH medium (A), ML medium (B), and MH medium compared to ML medium (C) at 24, 48, and 96 h. *, *P* < 0.05.

The metabolomics data revealed that levels of arginine and its related amino acids differed noticeably between MH and ML media (Table S1). Transcriptome analysis also showed that rich organic nitrogen significantly affected the expression levels of genes involved in arginine metabolism (Table S5). The arginine biosynthetic gene cluster was expressed at high levels during the first two stages of CA biosynthesis (at 24 and 48 h), with lower expression at 96 h in both MH and ML media (Fig. 5A and B). However, the expression levels of genes involved in arginine biosynthesis, especially the arginine biosynthetic gene cluster (*argJ*, *argB*, *argC*, *argD*, *argR*, *argG*, and *argH*), increased by 3- to 10-fold at 96 h in MH medium compared with those in ML medium (Fig. 5C). There were no significant differences in the expression levels of most of the arginine biosynthetic gene cluster at 24 and 48 h in MH and ML media (Table S5). RT-qPCR data also revealed that the expression trends of *argB*, *argC*, *argH*, and *argJ* were similar with that obtained from the transcriptome results (Fig. S2). Given that the arginine content in F613-1 was noticeably lower in MH medium than in ML medium (Fig. 3 and Table S1), our transcriptome and metabolome data collectively suggest that the conversion rate of arginine to CA is higher in MH medium than in ML medium.

### Enhanced amino acid metabolism promotes CA synthesis in MH medium.

**FIG 6:**
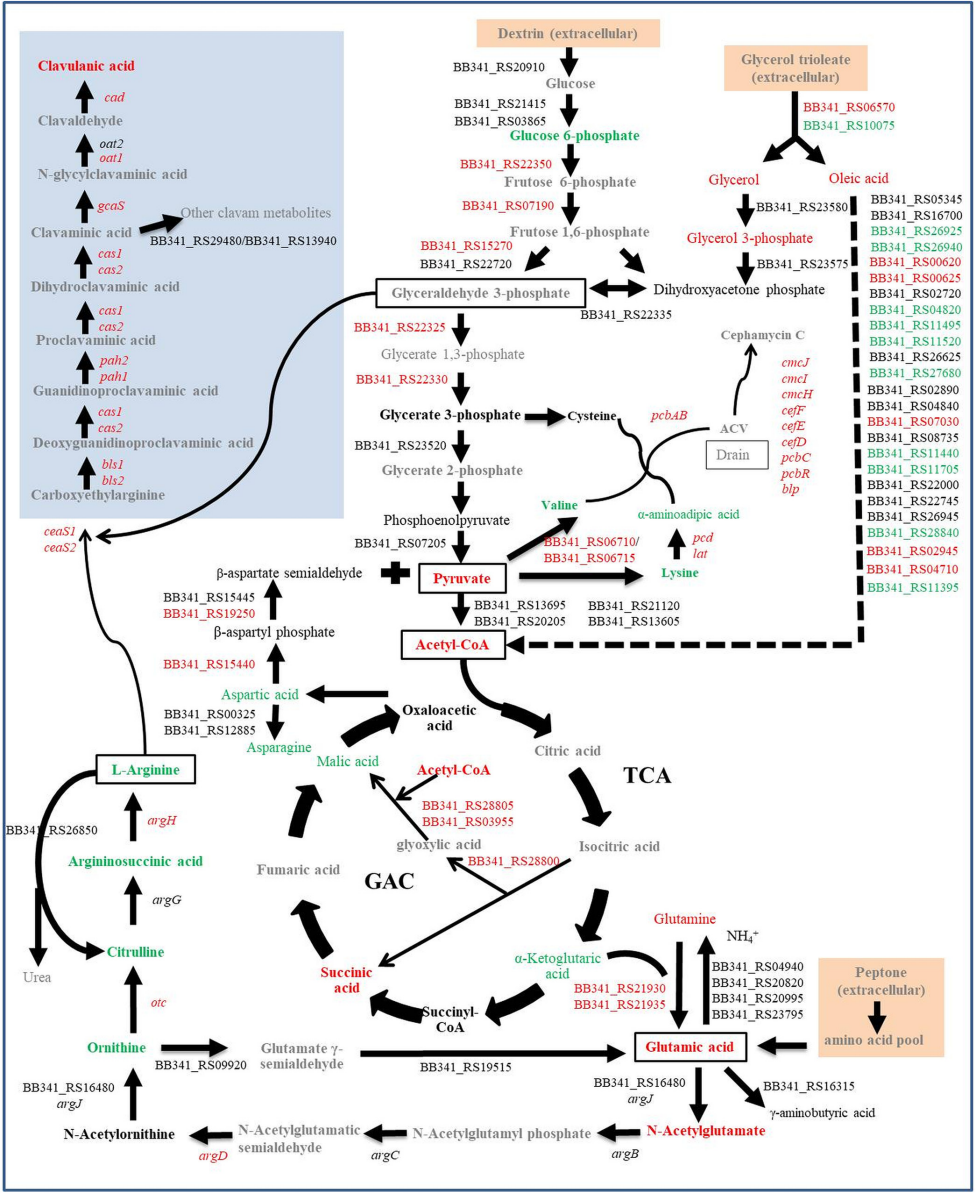
Schematic overview of correlation between metabolites and the expression profiles of genes involved in CA. Results for the metabolites and gene expression levels were obtained from the metabolome and transcriptome data for *S. clavuligerus* F613-1 cultured for 48 h (CA biosynthesis stage). Metabolites and genes shown in red were upregulated in MH medium compared to ML medium (MH48/ML48), those in green were downregulated (MH48/ML48), and those in black showed no change (MH48/ML48). Metabolites and genes shown in gray were not detected. TCA, tricarboxylic acid cycle; GAC, glyoxylic acid cycle.

To further evaluate how organic nitrogen affects CA biosynthesis, we comprehensively analyzed the transcriptome and metabolome of strain F613-1 cultured at 48 h (rapid stage of CA biosynthesis) in both MH and ML media and determined the metabolite and gene expression profiles involved in CA (Fig. 6). As shown in the schematic overview, all the genes associated with the CA biosynthesis pathway, including *cad*, *oat1*, *gcaS*, *cas1*, *cas2*, *pah2*, *pah1*, *ceaS1*, and *ceaS2*, were upregulated in MH medium compared with that in ML medium. Additionally, the level of intracellular metabolite l-glutamic acid was increased slightly in F613-1 at 48 h in MH medium, whereas the concentrations of arginine and its intermediates (argininosuccinic acid, citrulline, ornithine) decreased. Genes involved in converting glutamine into arginine, such as BB341_RS21930, BB341_RS21935, *argD*, *argR*, *argH*, and *otc*, were upregulated in F613-1 at 48 h in MH medium, indicating that arginine may be used in large quantities to synthesize CA. Results revealed that rich organic nitrogen enhances the CA biosynthesis pathway in MH medium compared with that in ML medium.

In addition, the cephalomycin C biosynthetic gene cluster (*pcd*, *cefE*, *cefD*, *cmcI*, *cmcJ*, *cefF*, *cmcH*, *ccaR*, *blp*, *lat*, *pcbAB*, *pcbC*, and *pcbR*) except for *pbpA* and *cmcT*, was notably upregulated in MH medium compared to that in ML medium (Table S7). The concentrations of the intracellular metabolites valine and α-aminoadipic acid, two precursors in cephalomycin C biosynthesis, were lower in MH medium than in ML medium (Fig. 6 and Table S1), and genes involved in converting pyruvate into valine and α-aminoadipic acid (such as BB341_RS06710, BB341_RS06715, *pcd*, and *lat*) were upregulated (Table S7). These results indicated that secondary metabolism in F613-1 was greatly influenced by organic nitrogen concentration.

### Supplementation with key amino acids promotes CA biosynthesis.

**FIG 7:**
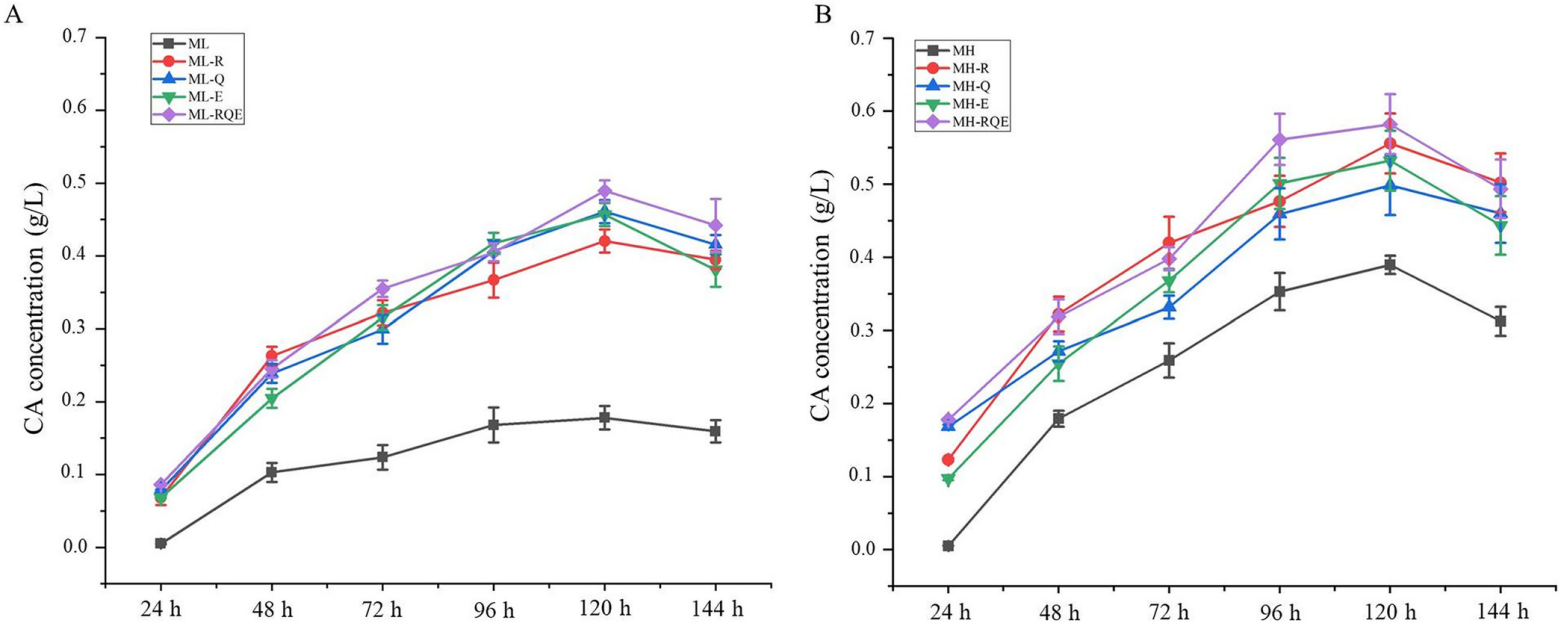
Effects of supplementation with arginine, glutamine, and glutamic acid on CA biosynthesis in F613-1. (A) Analysis of the changes in CA production in ML medium supplemented with 0.05% arginine (ML-R), 0.05% glutamine (ML-Q), 0.05% glutamic acid (ML-E), or a combination of 0.017% arginine, 0.017% glutamine, and 0.017% glutamic acid (ML-RQE). (B) Analysis of the changes in CA production in MH medium supplemented with amino acid as in ML medium. Data are the means ± SD of six separate experiments.

The above correlation analysis of metabolome and transcriptome data suggested that the concentrations of arginine, glutamine, and glutamic acid might directly affect CA biosynthesis in F613-1. To further evaluate their impact on CA fermentation, glutamine, glutamic acid, or arginine was added into ML and MH media to a final concentration of 0.05%. The CA concentration in F613-1 increased by about 3-fold in ML medium supplemented with any of the three amino acids at 120 h compared with that in ML medium. However, the effect of supplementation with arginine, glutamine, or glutamic acid on CA production in ML medium showed no statistical difference (Fig. 7A). The CA concentration in F613-1 increased by about 36% in MH medium supplemented with any of the three amino acids at 120 h compared with that in MH medium, and the impact of supplementation with arginine, glutamine, or glutamic acid on CA production in MH medium showed no statistical difference (Fig. 7B). In addition, the combination of arginine, glutamine, and glutamic acid could slightly increase CA production, but these differences were not statistically significant (Fig. 7). These results further indicate that supplementation of glutamine, glutamic acid, and arginine alone or a combination of these three amino acids could enhance CA biosynthesis.

## DISCUSSION

*S. clavuligerus* is the main producer of CA (7). Many studies have reported that inorganic nitrogen sources, such as sodium nitrate, ammonium carbonate, ammonium chloride, and potassium nitrate, do not affect CA biosynthesis or only slightly affect it (29), whereas CA biosynthesis is enhanced by organic nitrogen sources, such as soy protein and soy flour, that provide abundant C-5 precursors through the urea cycle (9, 29–31). However, supplementation with various amino acids showed inconsistent effects on CA production (29, 32–34). In this study, we found that MH and ML media, which contain different levels of peptone from soybean but are otherwise identical, had different effects on CA biosynthesis, with the richer levels of organic nitrogen in MH medium resulting in enhanced CA production. Both transcriptome and metabolome data revealed that organic nitrogen affected arginine metabolism, with decreases in the concentration of arginine and its intermediates (argininosuccinic acid, citrulline and ornithine) and increases in the concentration of glutamine and glutamic acid in MH medium (Table S1). Meanwhile, genes involved in converting glutamine into arginine were upregulated in MH medium (Table S5), indicating that high organic nitrogen may shift metabolism toward arginine synthesis, and then arginine may be used in large quantities to synthesize CA by providing the C-5 precursor for CA. Supplementation of ML medium with glutamic acid, arginine, or glutamine increased CA concentration in F613-1, further verifying that l-arginine is synthesized in the urea cycle, while l-glutamic acid and glutamine promote CA biosynthesis by fueling the urea cycle in the oxidative direction (31, 35). Overall, these data revealed that rich organic nitrogen or supplementation with glutamic acid, arginine, and glutamine can enhance CA production, and further illustrate that a constant supply of l-arginine is essential for CA biosynthesis.

G3P is the first C-3 precursor in CA biosynthesis (8, 9). Because glycerol acts as the C-3 provider, the addition of glycerol as well as glycerol trioleate can improve the production of CA in *S. clavuligerus* (9, 25, 29, 36). However, Lee and Ho reported that CA was not produced in medium with additional glycerol as the sole carbon source (37). Ives and Bushell also reported similar findings, with no CA production observed in glycerol-containing but carbon-limited medium (38). In this study, MH and ML media contained the same concentrations of dextrin and glycerol trioleate, carbon sources that did not appear to affect CA production under the tested conditions, as most of the intracellular carbohydrate content involved in the Embden-Meyerhof-Parnas pathway (EMP) and tricarboxylic acid cycle (TCA) was similar (Table S1). However, the intracellular glycerol content of F613-1 was higher in MH medium than in ML medium at 48 and 96 h (Fig. 3F), suggesting that glycerol might also be an important factor in the high CA yields in MH medium. Although Thakur et al. (39) reported that addition of glycerol or dextrin as a sole carbon source neither decreased nor improved CA production, it has also been reported that basal media containing glycerol exhibited higher maximum amounts of CA (9, 29), indicating that the role of glycerol in CA production requires further study.

Although metabolites in the EMP and TCA pathways exhibited no obvious changes during the rapid stage of CA biosynthesis (Table S1), many genes involved in converting glucose into glycerate-3-phosphate (such as BB341_RS22350, BB341_RS07190, and BB341_RS15270) and in converting G3P into acetyl coenzyme A (such as BB341_RS13605, BB341_RS13695, BB341_RS22330, BB341_RS22335, and BB341_RS21120) were obviously upregulated at 24 and/or 48 h in MH medium compared with levels in ML medium (Table S6). Genes involved in converting α-ketoglutaric acid into l-glutamic acid (such as BB341_RS21930 and BB341_RS21935) were also upregulated during the rapid stage of CA biosynthesis in MH medium (Table S5). These results indicate that the addition of rich organic nitrogen may influence carbon source metabolism, further impacting CA synthesis. In addition, the genes BB341_RS06710 and BB341_RS06715, which are involved in converting pyruvate into valine, the precursor of cephalomycin C, were also upregulated in MH medium (Table S7). This complicated network of secondary metabolites suggests that the role of carbon sources in CA biosynthesis requires further study.

Both the biomass and CA production of F613-1 in MH medium were higher than that in ML medium (Fig. 1), which may lead to a nutrient shortage and result in the decreased CA biosynthesis rate in MH medium during fermentation at 96 to 144 h. Key genes involved in CA biosynthesis (especially *gcaS*, a gene responsible for converting CA into *N*-glycyl-clavaminic acid) are downregulated at 96 h in MH medium compared with that in ML medium (Table S4 and Fig. 4), which may be caused by nutrient deficiency at 96 h in MH medium. Additionally, the expression levels of key genes involved in glyoxylic acid cycle and gluconeogenesis were sharply decreased at 96 h in MH medium compared with those in ML medium (Table S6), consistent with the greater decrease in reducing sugar in MH medium (Fig. 1), suggesting that the reducing sugar starvation may occur and therefore result in decreased CA biosynthesis rate and constant CA concentration between 96 and 144 h in MH medium.

GlnR is reported to activate nitrogen-assimilation genes under nitrogen-limiting conditions in *Streptomyces* (40, 41). The developmental regulator MtrA was reported to repress nitrogen-assimilation genes in nitrogen-rich media and compete with GlnR for binding to GlnR boxes in Streptomyces coelicolor (42). In this study, the transcriptome data revealed that expression of both *glnR* and *mtrA* was not affected in MH medium compared with that in ML medium at 24, 48, and 96 h in F613-1 (Table S5). The expression trends of both *glnR* and *mtrA* were decreased during the time evolution of the fermentation in MH medium (Table S2), but these differences were not statistically significant. The expression trend of *mtrA* was slightly decreased during the time evolution of the fermentation in MH medium (Table S2), and these differences were not statistically significant. The expression level of *glnR* was slightly decreased at 48 h (*P = *0.1891), but significantly decreased at 96 h (*P = *0.0017) in MH medium (Table S2). However, how the nitrogen metabolism genes are regulated and how the nitrogen metabolism affects CA biosynthesis require further study.

In conclusion, our findings indicate that optimization of organic nitrogen, arginine, glutamic acid, and glutamine levels can improve CA yields, which may be beneficial in industrial production. Additionally, given the importance of arginine metabolism in CA production, it may also be possible to genetically target arginine-associated pathways to engineer strains of *S. clavuligerus* with improved CA yields.

## MATERIALS AND METHODS

### Bacterial strains and media.

*S. clavuligerus* F613-1, an industrial strain for CA production (43, 44), was used in this study. Three types of culture medium were used in this study. Seed medium contained 2.0% peptone from soybean, 0.5% yeast extract, 1% soluble starch, and 0.08% dipotassium hydrogen phosphate, at pH 7.1. MH fermentation medium contained 0.8% peptone from soybean, 0.1% dextrin, 0.08% (vol/vol) glycerol trioleate, 0.15% potassium chloride, 0.1% magnesium chloride hexahydrate, 0.2% dipotassium hydrogen phosphate, 0.04% calcium chloride dihydrate, 0.008% ferric chloride hexahydrate, 0.001% zinc chloride, 0.018% sodium chloride, and 0.8% morpholinepropanesulfonic acid, at pH 7.1. The ML fermentation medium was identical to the MH medium except that it had a lower content of peptone from soybean: 0.4% instead of 0.8%.

### Fermentation conditions and sample treatment.

For F613-1 spore cultivation, CA fermentation was performed as described previously (25, 45). In brief, F613-1 spores (1 × 10^6^ CFU) were inoculated into 50 mL SCZ seed medium in 250-mL Erlenmeyer flasks and then cultured at 25°C with shaking at 200 rpm for 48 h to obtain seed cultures. Next, 2.5-mL seed cultures were centrifuged at 5,000 rpm to collect the mycelia, which were then transferred to 60 mL MH or ML medium in 250-mL Erlenmeyer flasks and grown at 25°C, with shaking at 200 rpm. Each fermentation experiment was repeated six times.

For analysis of CA, biomass, and reducing sugar concentration, 1-mL samples of fermentation liquid were collected at 24, 48, 72, 96, 120 and 144 h, and centrifuged at 5,000 rpm to collect the supernatant and pellets.

For detection of intracellular metabolites, 5-mL samples of fermentation liquid were harvested at 24, 48, and 96 h and centrifuged at 5,000 rpm to collect the mycelia, which were then transferred into new 2-mL tubes and washed three times with phosphate-buffered saline. The tubes were immediately immersed in liquid nitrogen and then frozen at −80°C until further analysis using ultra-high-performance liquid chromatography coupled with Q Exactive mass spectrometry (UHPLC-QE-MS) and ultra-high-performance liquid chromatography-quadrupole time-of-flight mass spectrometry (UHPLC-QTOF-MS). Six independent samples were prepared for each treatment.

To detect the transcriptome, 8-mL samples of fermentation liquid were harvested at 24, 48, and 96 h and centrifuged at 5,000 rpm to collect the mycelium, which was immediately immersed in liquid nitrogen and then frozen at −80°C. Three independent samples were prepared for each treatment.

### Analysis of CA and biomass.

CA concentration in the fermentation supernatants and the biomass in the fermentation broth at different time points from MH and ML fermentation were measured as previously described (25).

### Determination of the reducing sugar concentration.

The fermentation supernatants, harvested at different time points from MH and ML fermentation, were filtered using a 0.22-μm membrane. The reducing sugar concentrations in the fermentation supernatants were determined using the Reducing Sugar Content Assay kit (Boxbio, China).

### Intracellular metabolite extraction.

Intracellular metabolites were extracted using previously described methods (46) with some modifications. For the extractions, each liquid nitrogen-quenched sample was homogenized using a Beadbug homogenizer (Benchmark Scientific, NJ, USA). Next, 400 μL extraction solution (methanol:acetonitrile = 1:1 [vol/vol], including an isotope-labeled internal standard mixture), was added into each sample, followed by vortexing for 30 s, ultrasonication for 10 min in an ice water bath, standing for 1 h at −40°C, and then centrifugation at 4°C, 12,000 rpm for 15 min. Finally, the supernatants were collected for UHPLC-QE-MS detection.

### UHPLC-QE-MS detection for intracellular metabolites.

UHPLC-QE-MS detection for intracellular metabolites was performed by Biotree Biomedical Technology Co., Ltd. (Shanghai, China), using a UHPLC system (Vanquish, Thermo Fisher Scientific, MA, USA) with a UPLC ethylene bridged hybrid (BEH) amide column (2.1 × 100 mm, 1.7 μm) coupled to a Q Exactive HFX mass spectrometer (Orbitrap MS, Thermo Fisher Scientific). Mobile phase A (25 mmol/L ammonium acetate and 25 mmol/L ammonia hydroxide in water [pH 9.75]) and mobile phase B (100% acetonitrile) were used for both electrospray ionization (ESI) positive and negative modes. The ESI source conditions were set as follows: sheath gas flow rate, 30 Arb; aux gas flow rate, 25 Arb; capillary temperature, 350°C; full MS resolution, 60,000; tandem mass spectrometry (MS/MS) resolution, 7,500; collision energy, 10/30/60 in NCE mode; and spray voltage, 3.6 kV (positive) or −3.2 kV (negative).

### Intracellular lipidome metabolite extraction.

Each liquid nitrogen-quenched sample was transferred into a 280-μL solution of water:methanol (5:2). Each sample was homogenized at 35 Hz for 4 min and ultrasonicated for 5 min in an ice-water bath; both the homogenization and ultrasonication cycles were repeated three times. Equivalent amounts of homogenate based on weight were taken, and water was added to give a final volume of 280 μL. Next, 400 μL methyl tert-butyl ether was added to each sample, followed by vortexing for 60 s and sonication for 10 min in an ice-water bath. Samples were incubated at −40°C for 1 h and centrifuged at 3,000 rpm for 15 min at 4°C, and 350 μL of each supernatant was transferred to a fresh tube and dried in a vacuum concentrator at 37°C. The dried samples were reconstituted in 100 μL solution (dichloromethane:methanol = 1:1) by sonication on ice for 10 min. The reconstituted samples were then centrifuged at 4°C at 13,000 rpm for 15 min, and 75 μL supernatant was transferred to a fresh glass vial for UHPLC-QTOF-MS analysis.

### UHPLC-QTOF-MS for intracellular lipidome metabolites.

UHPLC-QTOF-MS detection of intracellular lipidome metabolites was performed by Biotree Biomedical Technology Co., Ltd. (Shanghai, China), using a 1290 Infinity series UHPLC System (Agilent Technologies, CA, USA), equipped with a Kinetex C_18_ column (2.1 × 100 mm, 1.7 μm, Phenomenex, CA, USA). Mobile phase A was 40% water, 60% acetonitrile, and 10 mmol/L ammonium formate; and mobile phase B was 10% acetonitrile, 90% isopropanol, and 0.5 mmol/L ammonium formate. The elution gradient was set as follows: 0 ~ 12.0 min, 40% ~ 100% B; 12.0 ~ 13.5 min, 100% B; 13.5 ~ 13.7 min, 100% ~ 40% B; 13.7 ~ 18.0 min, 40% B. The column temperature was 45°C and the auto-sampler temperature was 4°C. The injection volume was 2 μL (positive mode ESI-MS/MS) or 6 μL (negative mode ESI-MS/MS). A TripleTOF mass spectrometer was used for its ability to acquire MS/MS spectra on an information-dependent basis during a liquid chromatography-mass spectrometry (LC/MS) experiment.

### Data preprocessing and statistical analysis.

The raw data from UHPLC-QE-MS were converted to the mzXML format using ProteoWizard and processed with an in-house program for peak detection, extraction, alignment, and integration. Next, an in-house MS2 database (BiotreeDB) was applied for metabolite annotation. The cutoff for annotation was set at 0.3 (Biotree, China).

The raw data files (.wiff format) from UHPLC-QTOF-MS were converted to files in mzXML format using the ‘msconvert’ program from ProteoWizard. Peak detection was first applied to the MS1 data. The CentWave algorithm in XCMS was used for peak detection, with the MS/MS spectra, and lipid identification was achieved through a spectral match using an in-house MS/MS spectral library (Biotree, China).

Statistical analysis was performed as previously described (47). Supervised OPLS-DA was carried out to visualize group separation and find significantly changed metabolites. Furthermore, the values for VIP of the first principal component in OPLS-DA analysis were obtained, and metabolites with a VIP of >1 and *P* < 0.05 (Student’s *t* test) were selected as the key metabolites.

### Transcriptome sequencing and analysis.

Total RNA of each sample was extracted and treated according to the recommended protocols (25). The transcriptome was sequenced using a HiSeq 3000 sequencer (Illumina, CA, USA) and preliminarily analyzed at Biotree Corporation (Shanghai, China). The expression level of each gene was normalized by the number of reads per kilobase per million mapped reads. The differentially expressed genes were selected using the DESeq program the parameters |log2FoldChange| > 1 and *P* < 0.05. GO (Gene Ontology, http://geneontology.org/) and KEGG (Kyoto Encyclopedia of Genes and Genomes, http://www.kegg.jp/) analyses were performed to construct the pathway associated with CA biosynthesis.

### Verification of the effect of key metabolites on CA production.

Spores of F613-1 were inoculated into seed medium and then cultured at 25°C, 200 rpm, for 48 h to obtain seed cultures. Aliquots of the seed cultures were transferred to the following media (i): ML-R, containing ML fermentation medium and 0.05% arginine; (ii) ML-Q, containing ML fermentation medium and 0.05% glutamine; (iii) ML-E, containing ML fermentation medium and 0.05% glutamic acid; (iv) ML-RQE, containing ML fermentation medium and 0.017% arginine, 0.017% glutamine, and 0.017% glutamic acid; (v) MH-R, containing MH fermentation medium and 0.05% arginine; (vi) MH-Q, containing MH fermentation medium and 0.05% glutamine; (vii) MH-E, containing MH fermentation medium and 0.05% glutamic acid; and (viii) MH-RQE, containing MH fermentation medium and 0.017% arginine, 0.017% glutamine, and 0.017% glutamic acid. The CA yields from the fermentation broth were measured at 24, 48, 72, 96, 120, and 144 h using HPLC as indicated in the fermentation experiments described above. All statistical analyses were performed using GraphPad Prism 9.0 software.

### Real-time quantitative PCR analysis.

Real-time quantitative PCR assays were performed as previously described (48) with some modifications. Mycelia of *S. clavuligerus* F613-1 were harvested from MH and ML fermentation liquid at 24, 48, and 96 h and rapidly frozen in liquid nitrogen, and then total RNA was extracted using a Bacteria Total RNA Isolation kit (Sangon Biotech, Shanghai, China). To remove the residual chromosomal DNA, total RNA samples were treated with RNase-free DNase I (Sigma-Aldrich, MO, USA). Next, the cDNAs were synthesized using random hexamer primers (Sigma-Aldrich), M-MLV reverse transcriptase (Invitrogen), and deoxynucleoside triphosphates (Sigma-Aldrich). RT-qPCR assays were performed on the Roche LightCycler 480 using SYBR Premix *Ex Taq* kit (TaKaRa Bio, Japan). Relative quantities of cDNA were normalized to the amounts of 16S rDNA. For RT-qPCR assays, experiments were conducted in three independent replications. Primers used for RT-qPCR are listed in Table S8 in the supplemental material. Statistical analyses were performed using GraphPad Prism 9.0 software.

### Data availability.

Transcriptomic data have been assigned the GEO accession number GSE211765. Metabolomics data have been deposited to the EMBL-EBI MetaboLights database with the identifier MTBLS5744.
