# Supplementary material for: Rich Organic Nitrogen Impacts Clavulanic Acid Biosynthesis through the Arginine Metabolic Pathway in Streptomyces clavuligerus F613-1
Source: Microbiol Spectr. 2022 Dec 14;11(1):e02017-22. doi: 10.1128/spectrum.02017-22 (PMC9927107; doi:10.1128/spectrum.02017-22)
Supplement: Supplemental file 1 — Supplemental material. Download spectrum.02017-22-s0001.pdf, PDF file, 0.5 MB [file spectrum.02017-22-s0001.pdf]

Supplementary Material

Rich organic nitrogen impacts clavulanic acid biosynthesis through the arginine metabolic pathway in *Streptomyces clavuligerus* F613-1

Jiafang Fu [a,b], Xinru Xie [a], Shaowei Zhang [a], Ni Kang [a], Gongli Zong [a,b], Peipei Zhang [a,b], Guangxiang Cao [a,b#]

[a] Biomedical Sciences College & Shandong Medicinal Biotechnology Centre, Shandong First Medical University & Shandong Academy of Medical Sciences, Jinan 250117, China

[b] NHC Key Laboratory of Biotechnology Drugs, Shandong Academy of Medical Sciences, Jinan 250117, China

# Corresponding author: Guangxiang Cao, Biomedical Sciences College, Shandong First Medical University & Shandong Academy of Medical Sciences, Qingdao Road 6699, Jinan 250117, Shandong, P.R. China. Tel.: +86 531 59567322, E-mail: caoguangxiang@sdfmu.edu.cn

Table S1 Comparison of intracellular metabolites concentration in MH and ML medium.

Metabolites	Growth phase (24 h)		Growth phase (48 h)		Growth phase (96 h)	
	ML/MH[a]	VIP[b]	ML/MH[a]	VIP[b]	ML/MH[a]	VIP[b]
**EMP**						
Glucose 6-phosphate	7.25/6.10	0.38	8.11/7.19	0.42	8.31/14.81	1.00
Pyruvate	7.81/8.89	0.58	5.47/8.03	1.31	6.97/3.85	1.03
**TCA**						
L-Malic acid	11.97/8.00	0.71	23.42/17.76	0.55	44.30/129.02	1.05
Succinic acid	14.61/14.63	0.20	13.79/15.89	0.98	16.12/11.74	1.16
α-Ketoglutaric acid	19.18/20.07	0.43	27.05/21.48	0.80	39.86/54.42	1.13
**Lipids**						
TG	1135.82/1842.93	1.42	1165.82/2060.24	1.49	1164.83/1815.89	1.10
DG	83.53/264.66	1.52	97.35/146.32	1.23	50.95/154.48	1.61
MG	5.60/7.56	1.23	7.01/4.77	0.94	11.81/6.29	1.36
Oleic acid	2705.65/2453.84	0.46	2129.49/2340.76	0.95	1484.87/1184.68	0.58
Glycerol	3.54/3.71	0.12	3.99/6.94	1.59	2.90/3.43	1.12
**Amino acids**						
L-Arginine	48.07/26.41	1.21	65.58/46.64	1.27	49.18/33.26	1.24
L-Glutamic acid	68.24/72.87	1.16	59.62/67.32	0.53	82.80/46.43	1.05
L-Glutamine	617.29/643.05	0.67	736.18/1013.73	1.54	1140.53/1293.58	0.97
L-Lysine	19.19/14.91	1.00	24.69/17.92	0.96	25.27/39.31	1.03
L-Valine	1365.26/1250.88	0.54	1533.78/1151.41	1.27	1446.53/1879.56	1.09
L-Aspartic acid	258.74/249.34	0.12	385.63/253.31	1.15	344.16/508.72	1.12
L-Asparagine	155.15/112.79	0.87	191.25/121.10	1.16	135.43/186.80	1.14
Argininosuccinic acid	0.90/0.56	1.21	2.86/1.54	1.20	6.52/5.91	0.17
Ornithine	2.10/1.07	1.41	6.725/4.68	1.19	7.58/20.37	1.53
N-Acetylornithine	4.78/5.90	0.60	3.13/3.86	0.66	12.24/3.45	0.91
Citrulline	2.73/1.90	1.50	5.20/3.55	0.55	11.93/10.83	0.42
N-Acetylglutamic acid	15.01/17.81	1.29	7.99/15.93	1.72	28.49/17.14	1.19
**Others**						
Terephthalic acid	23.07/29.13	1.45	17.63/22.21	0.82	17.36/12.31	0.84
gamma-Aminobutyric acid	430.49/815.96	1.77	117.93/123.22	0.22	101.78/76.24	1.10
α-aminoadipic acid	6.97/13.78	1.77	31.85/12.84	1.42	60.61/130.41	1.18
Diaminopimelic acid	8.36/12.10	1.39	5.17/16.55	1.49	22.64/31.77	0.86

Note: This table contained data from UHPLC-QE-MS (negative), UHPLC-QE-MS (positive), and UHPLC-QTOF-MS (positive). [a] The concentration of metabolites was presented by their relative abundance as the average of six independent experiments. [b] Variable Importance in the Projection (VIP) scores, metabolites with VIP score > 1 were considered as the key metabolite closely related to CA production.

Table S2 Comparison of the transcriptional level of genes at different time points in MH medium.

genes	log2Fold Change ratio			Description
	MH48/MH24	MH96/MH24	MH96/MH48	
**CA biosynthetic gene cluster**				
ceaS2	-0.324649177	-5.533093947	-5.183900668	Carboxyethylarginine synthase
bls2	0.062921499	-4.197252054	-4.245823245	conserved hypothetical protein
pah2	0.203722879	-4.79564716	-4.987110522	proclavaminate amidino hydrolase, PAH
gas2	0.241441695	-4.749810993	-4.968983253	Clavaminate synthase 2
oat2	0.392904738	-2.024568446	-2.399144279	Glutamate N-acetyltransferase 2 beta chain
oppA1	-0.192070544	-2.992050628	-2.787340039	ABC-type dipeptide transport system, periplasmic component
claR	-1.695896373	-3.58824332	-1.879779805	conserved hypothetical protein
cad	-0.998754081	-4.806246741	-3.78101082	conserved hypothetical protein
cyp450	-0.976561228	-3.208422186	-2.204467432	cytochrome P450-SU2
fd	-0.504735822	-3.785264398	-3.227587556	Ferredoxin
Orf12	-0.770387108	-4.010445574	-3.209011829	unknown
Orf13	-0.466318774	-2.003222213	-1.481320496	conserved hypothetical protein
Orf14	0.362173514	-2.426803733	-2.770468426	conserved hypothetical protein
oppA2	0.393862529	-3.20878078	-3.5633271	ABC-type dipeptide transport system, solute-binding protein
Orf16	0.120960691	-3.450872789	-3.541757716	conserved hypothetical protein
gcaS	-0.243832548	-4.330900184	-4.059483853	biotin carboxylase
pbps	-0.393690134	-1.450479041	-1.029191263	penicillin-binding protein PBP
pbps	0.170352247	-0.611536293	-0.75249859	Penicillin-binding protein
cyp	-0.313969407	0.559981583	0.900016756	cytochrome P450 hydroxylase
cagS	-0.60477741	-0.605015616	0.008967954	ATPase domain-containing protein
cagR	1.631724893	1.694900945	0.081065767	two-component system response regulator
**paralogue gene cluster**				
ceaS1	-0.278320191	-5.236610103	-4.920054002	carboxyethyl arginine synthase isoenzyme 1
bls1	-0.377882692	-5.731199049	-5.329627348	Beta-lactam synthetase isoenzyme 1
pah1	-0.518511797	-6.137579996	-5.596842328	proclavaminate amidinohydrolase 1
oat1	0.425859077	-3.586541501	-3.999828325	Probable glutamate N-acetyltransferase 1
**clavam gene cluster**				
cvmG	2.259261732	2.085888941	-0.059770029	hypothetical protein
cvmP	-1.628063819	-4.687960038	-3.046477495	putative protein-arginine deiminase
cvmH	1.972825676	1.112121685	-0.74828356	LanU-like protein
cvm13	0.53429108	-0.787073163	-1.304174614	Beta-aspartyl-peptidase
cvm7	-1.785826951	-1.759015205	-0.960573926	DnrI/RedD/AfsR-family transcriptional regulator
cvm3	-4.881498971	-5.187752832	-2.526651011	flavin reductase-like protein
cvm2	-5.454903722	-5.166361686	-3.744831749	Hypothetical protein
cvm1	-4.462887356	-4.048234579	-1.756108608	Aldo/keto reductase
cas1	-2.104899334	-2.982986552	-3.631037819	Clavaminate synthase 1

cvm4	-5.895257195	-5.20240858	-2.771700275	Alpha/beta hydrolase
cvm5	-6.630947728	-5.559162274	-3.671693282	Flavin-dependent oxidoreductase, F420-dependent methylene-tetrahydromethanopterin reductase
cvm6	-3.8297128	-1.405779568	0.218604782	Putative pyridoxal phosphate-dependent aminotransferase
**Arginine biosynthesis gene cluster**				
argJ	0.53307611	-2.594956429	-3.089751189	arginine biosynthesis bifunctional protein argJ
argB	0.706310661	-2.273770535	-2.948126301	acetylglutamate kinase
argC	0.770381426	-1.330607117	-2.082800202	N-acetyl-gamma-glutamyl-phosphate reductase
argD	0.881695216	-2.513775636	-3.366300001	acetylornithine aminotransferase
BB341_RS04845	-0.327186056	-2.819320041	-2.460065623	Ornithine carbamoyltransferase
argR	0.748822091	-2.553193257	-3.279076515	arginine repressor
argG	-0.175474746	-3.065670073	-2.861478545	Argininosuccinate synthase
argH	0.091623665	-3.126081438	-3.183020841	argininosuccinate lyase
BB341_RS26850	-2.393983324	-2.281831269	0.136720419	conserved hypothetical protein
BB341_RS09920	1.414333258	0.977975875	-0.404619156	Putative ornithine aminotransferase
**Nitrogen-assimilation regulating genes**				
gene-BB341_RS12660 (*glnR*)	-0.166353	-0.968822	-0.787555	transcriptional regulatory protein GlnR
gene-BB341_RS18105 (*mtrA*)	-1.582546	-0.94684	0.6466482	DNA-binding response regulator MtrA
**Key genes involved in glyoxylic acid cycle and gluconeogenesis**				
BB341_RS28800	7.06557453	-0.33177041	-7.378360769	Isocitrate lyase
BB341_RS28805	5.30752461	0.865655253	-4.42211839	Malate synthase
BB341_RS03955	3.38173010	1.320714347	-2.048584246	Malate synthase
BB341_RS09375	2.88502592	-1.870553666	-4.776907211	phosphoenolpyruvate carboxykinase
BB341_RS08985	1.63103971	-1.048884908	-2.659692474	fructose 1,6-bisphosphatase II
BB341_RS08395	-1.0707528	-1.656750232	-0.570597353	fructose-1,6-bisphosphatase
BB341_RS14335	0.05673254	-0.464160468	-0.4244792492	Sugar phosphatase
BB341_RS28280	0.091853062	-4.981771072	-5.037702203	Phosphoenolpyruvate synthase

Note: Red lettering indicates up-regulated genes. Green lettering indicates down-regulated genes.

Table S3 Comparison of the transcriptional level of genes at different time points in ML medium.

genes	log2Fold Change ratio			Description
	ML48/ML24	ML96/ML24	ML96/ML48	
**CA biosynthetic gene cluster**				
ceaS2	-4.480077236	-4.588318466	-0.110290942	Carboxyethylarginine synthase
bls2	-3.416110496	-3.283903484	0.128228637	conserved hypothetical protein
pah2	-3.508103426	-3.741355686	-0.237460113	proclavaminate amidino hydrolase, PAH
gas2	-3.573609147	-3.733566961	-0.163944404	Clavaminate synthase 2
oat2	-0.208732464	-5.283404887	-5.081331214	Glutamate N-acetyltransferase 2 beta chain
oppA1	-0.849270478	-3.88229129	-3.035741061	ABC-type dipeptide transport system, periplasmic component
claR	-1.712962049	-1.541225353	0.171591789	conserved hypothetical protein
cad	-3.846505341	-3.225918961	0.619925819	conserved hypothetical protein
cyp450	-1.989227574	-1.399144572	0.59145995	cytochrome P450-SU2
fd	-3.650444951	-2.325075123	1.328816238	Ferredoxin
Orf12	-2.845294898	-2.213323045	0.631223623	unknown
Orf13	-0.753029516	-0.732933704	0.02343436	conserved hypothetical protein
Orf14	-1.583915254	-0.794060939	0.791334157	conserved hypothetical protein
oppA2	-2.898374771	-1.585054817	1.313389867	ABC-type dipeptide transport system, solute-binding protein
Orf16	-3.452734145	-2.230792056	1.22156962	conserved hypothetical protein
gcaS	-3.312779136	-2.091872393	1.219954858	biotin carboxylase
pbps	-1.330059243	-1.589660891	-0.263036685	penicillin-binding protein PBP
pbps	-0.520125305	-0.935543902	-0.418871682	Penicillin-binding protein
cyp	0.767507785	1.195483835	0.426979773	cytochrome P450 hydroxylase
cagS	-0.271654752	-0.496791865	-0.225664891	ATPase domain-containing protein
cagR	0.628467682	0.865041817	0.230912167	two-component system response regulator
**paralogue gene cluster**				
ceaS1	-2.291090007	-6.14166493	-3.857979876	carboxyethyl arginine synthase isoenzyme 1
bls1	-2.776997903	-6.64004797	-3.868884756	Beta-lactam synthetase isoenzyme 1
pah1	-2.714625351	-6.698780623	-3.992392402	proclavaminate amidinohydrolase 1
oat1	-1.352455205	-4.137569367	-2.787364965	Probable glutamate N-acetyltransferase 1
**clavam gene cluster**				
cvmG	0.49836631	0.876893948	0.379886926	hypothetical protein
cvmP	-1.030208181	-1.529037758	-0.499406969	putative protein-arginine deiminase
cvmH	0.27244818	-0.080552254	-0.351289963	LanU-like protein
cvm13	-0.015511608	-0.866643674	-0.85078159	Beta-aspartyl-peptidase
cvm7	-0.584732287	-1.543911672	-0.960573926	DnrI/RedD/AfsR-family transcriptional regulator
cvm3	-2.601796683	-5.126998462	-2.526651011	flavin reductase-like protein
cvm2	-2.121524225	-5.856796978	-3.744831749	Hypothetical protein
cvm1	-2.603622486	-4.357513514	-1.756108608	Aldo/keto reductase
cas1	-0.370373546	-3.995150128	-3.631037819	Clavaminate synthase 1

cvm4	-2.802469164	-5.569157818	-2.771700275	Alpha/beta hydrolase
cvm5	-2.770144843	-6.433314788	-3.671693282	Flavin-dependent oxidoreductase, F420-dependent methylene-tetrahydromethanopterin reductase
cvm6	-3.288339182	-3.067476227	0.218604782	Putative pyridoxal phosphate-dependent aminotransferase
**Arginine biosynthesis gene cluster**				
argJ	0.597471676	-4.539047675	-5.142315569	arginine biosynthesis bifunctional protein argJ
argB	0.75808054	-4.027501261	-4.787859072	acetylglutamate kinase
argC	0.434773104	-4.099041809	-4.536607531	N-acetyl-gamma-glutamyl-phosphate reductase
argD	0.481980629	-4.876169151	-5.365023876	acetylornithine aminotransferase
BB341_RS04845	-0.989098482	-1.744732839	-0.755178236	Ornithine carbamoyltransferase
argR	0.439784231	-4.404117503	-4.847136907	arginine repressor
argG	-0.582866056	-5.410274939	-4.834891607	Argininosuccinate synthase
argH	-0.306934336	-3.97175008	-3.667788865	argininosuccinate lyase
BB341_RS26850	-4.025908521	-3.167413724	0.859944435	conserved hypothetical protein
BB341_RS09920	1.780757113	0.586896067	-1.197705263	Putative ornithine aminotransferase
**Nitrogen-assimilation regulating genes**				
gene-BB341_RS12660 (*glnR*)	-0.406902	-1.227523	-0.824656	transcriptional regulatory protein GlnR
gene-BB341_RS18105 (*mtrA*)	-0.66321	-1.046232	-0.385663	DNA-binding response regulator MtrA
**Key genes involved in glyoxylic acid cycle and gluconeogenesis**				
BB341_RS28800	0.313520631	0.448747007	0.133550724	Isocitrate lyase
BB341_RS28805	1.076322737	1.63386155	0.559761227	Malate synthase
BB341_RS03955	2.291737339	0.229240639	-2.064084037	Malate synthase
BB341_RS09375	0.871842621	-2.808424747	-3.687330111	phosphoenolpyruvate carboxykinase
BB341_RS08985	0.78902119	-0.860533771	-1.656397054	fructose 1,6-bisphosphatase II
BB341_RS08395	-0.316733314	-0.662489268	-0.34981003	fructose-1,6-bisphosphatase
BB341_RS14335	-0.064465676	0.326929127	0.260479242	Sugar phosphatase
BB341_RS28280	-3.196374003	-4.760772201	-1.568930189	Phosphoenolpyruvate synthase

Note: Red lettering indicates up-regulated genes. Green lettering indicates down-regulated genes

Table S4 Comparison of transcriptional level of genes involved in CA biosynthesis in MH and ML medium.

genes	Log2(Fold Change) ratio			Description
	MH24/ML24	MH48/ML48	MH96/ML96	
**CA biosynthetic gene cluster**				
ceaS2	1.010991507	5.12852479	0.027709783	Carboxyethylarginine synthase
bls2	0.951701927	4.402072219	-0.001009081	conserved hypothetical protein
pah2	0.627809993	4.31202595	-0.465625188	proclavaminate amidino hydrolase, PAH
cas2	0.748081966	4.528941545	-0.308948195	Clavaminate synthase 2
oat2	0.397955348	0.962288723	3.617474099	Glutamate N-acetyltransferase 2 beta chain
oppA1	0.828357798	1.453279264	1.676275444	ABC-type dipeptide transport system, periplasmic component
claR	1.906056429	1.888706247	-0.188421345	conserved hypothetical protein
cad	1.364717875	4.173193244	-0.261176402	conserved hypothetical protein
cyp450	1.604763856	2.568680926	-0.249847968	cytochrome P450-SU2
fd	1.030566219	4.093572249	-0.465062774	Ferredoxin
Orf12	1.310081655	3.342374897	-0.529646275	unknown
Orf13	1.222448479	1.431141765	-0.093106979	conserved hypothetical protein
Orf14	1.018959028	2.928427588	-0.661061794	conserved hypothetical protein
oppA2	0.811908782	4.054644016	-0.843601929	ABC-type dipeptide transport system, solute-binding protein
Orf16	0.864965454	4.401595593	-0.389171018	conserved hypothetical protein
gcaS	1.287324501	4.312328533	-0.994631503	biotin carboxylase
pbps	0.553691881	1.449151842	0.645760309	penicillin-binding protein PBP
pbps	0.597881177	1.245010041	0.874816761	Penicillin-binding protein
cyp	0.251248276	-0.864865411	-0.422620814	cytochrome P450 hydroxylase
cagS	0.130722725	-0.229668029	-0.024849616	ATPase domain-containing protein
cagR	-1.327032874	-0.358464346	-0.549360173	two-component system response regulator
**Paralogue gene cluster**				
ceaS1	-0.107182531	1.854741564	0.35719405	carboxyethyl arginine synthase isoenzyme 1
bls1	0.013945955	2.374427513	0.322940937	Beta-lactam synthetase isoenzyme 1
pah1	-0.194289859	1.967785896	0.611756494	proclavaminate amidinohydrolase 1
oat1	-0.118908908	1.628399897	0.254094039	Probable glutamate N-acetyltransferase 1
**Clavam gene cluster**				
cvmG	-1.477743756	0.177571009	-0.300155732	hypothetical protein
cvmP	3.490248237	2.870554613	0.297886194	putative protein-arginine deiminase

cvmH	-0.990560959	0.59888885	0.172227351	LanU-like protein
cvm13	-0.223284119	0.280592963	-0.20589045	Beta-aspartyl-peptidase
cvm7	0.399534983	0.939353011	0.106231445	DnrI/RedD/AfsR-family transcriptional regulator
cvm3	0.12163333	3.017676061	0.321354906	flavin reductase-like protein
cvm2	0.003173252	1.821378139	0.361638599	Hypothetical protein
cvm1	0.255557764	2.427985006	0.102193118	Aldo/keto reductase
cas1	0.019799804	1.248384708	1.870810797	Clavaminate synthase 1
cvm4	0.27717079	2.374884141	-0.091475418	Alpha/beta hydrolase
cvm5	0.300000628	1.987808836	0.065102614	Flavin-dependent oxidoreductase, F420-dependent methylene-tetrahydromethanopterin reductase
cvm6	0.463767041	1.31395106	-0.342049366	Putative pyridoxal phosphate-dependent aminotransferase

Note: Red lettering indicates up-regulated genes. Green lettering indicates down-regulated genes.

Table S5 Comparison of transcriptional level of genes involved in amino acids metabolism in MH and ML medium.

genes	log2Fold Change ratio			Description
	MH24/ML24	MH48/ML48	MH96/ML96	
**Arginine metabolism**				
BB341_RS21930	0.876218	1.3153591	0.426599	Glutamate synthase
BB341_RS21935	1.093263	1.740667	0.753968	Glutamate synthase subunit beta
BB341_RS04940	-0.32942	0.3382503	-0.37394	L-glutamine synthetase
BB341_RS20820	0.46901	0.563109	0.667911	glutamine synthetase
BB341_RS20995	0.58218	-0.323331	-0.26023	Glutamine synthetase 2
BB341_RS23795	-0.06064	0.2481738	0.37563	Putative glutamine synthetase
BB341_RS16315	-1.54171	-0.200255	-0.34464	glutamate decarboxylase
BB341_RS16480	0.41204	-0.737814	0.254692	N-acetylglutamate synthase
BB341_RS23950 (*argJ*)	0.787829	0.676534	2.705063	arginine biosynthesis bifunctional protein argJ
BB341_RS23955 (*argB*)	0.996505	0.9024931	2.718168	acetylglutamate kinase
BB341_RS23945 (*argC*)	0.525775	0.8332233	3.26456	N-acetyl-gamma-glutamyl-phosphate reductase
BB341_RS23960 (*argD*)	1.00352	1.3667261	3.341257	acetylornithine aminotransferase
BB341_RS23965 (*argR*)	0.776484	1.0523178	2.595821	arginine repressor
BB341_RS23970 (*argG*)	0.222048	0.5871702	2.531078	Argininosuccinate synthase
BB341_RS23975 (*argH*)	0.915324	1.2746741	1.732178	argininosuccinate lyase
BB341_RS26850	-0.61497	0.9667093	0.219634	conserved hypothetical protein
BB341_RS09920	0.103372	-0.307784	0.448394	Putative ornithine aminotransferase
BB341_RS04845 (*otc*)	1.136973	1.7531363	0.020208	Ornithine carbamoyltransferase
BB341_RS19515	0.568971	0.061634	0.299051	gamma-glutamyl phosphate reductase
gene-BB341_RS12660 (*glnR*)	0.4513483	0.6546325	0.6546981	transcriptional regulatory protein GlnR
gene-BB341_RS18105 (*mtrA*)	0.4829468	-0.447992	0.5491339	DNA-binding response regulator MtrA

Note: Red lettering indicates up-regulated genes. Green lettering indicates down-regulated genes.

Table S6 Comparison of transcriptional level of genes involved in lipid and carbohydrate metabolism in MH and ML medium.

genes	log2Fold Change ratio			Description
	MH24/ML24	MH48/ML48	MH96/ML96	
**Glycerol trioleate converted in to glycerol**				
BB341_RS06570	-1.0892002	3.89731069	0.688892	Putative secreted lipase
BB341_RS10075	-0.3338632	-1.4452148	0.833921	lipase
BB341_RS23580	0.27801735	0.71034635	0.366114	glycerol kinase
BB341_RS23575	-0.5055819	-0.1264923	-0.23426	Glycerol-3-phosphate dehydrogenase
BB341_RS05345	-0.2825334	-0.9700032	-0.23687	Putative acyl-CoA synthetase
BB341_RS16700	-0.3874935	-0.3278471	0.467232	AMP-dependent synthetase and ligase
BB341_RS26925	-1.3321493	-1.7717991	0.415322	Putative acyl-CoA synthetase, long-chain fatty acid:CoA ligase
BB341_RS26940	-2.3899957	-1.6285275	-0.4338	FadD-like protein
BB341_RS00620	-0.0560955	2.20483364	0.201387	Acyl-CoA dehydrogenase
BB341_RS00625	0.02416384	1.6199683	-0.03156	acyl-CoA dehydrogenase
BB341_RS02720	-1.3772325	-0.3453542	0.188589	Probable acyl-CoA dehydrogenase
BB341_RS04820	-0.4460056	-1.196256	0.665203	acyl-CoA dehydrogenase
BB341_RS11495	0.08124229	-1.6527805	-0.40725	Putative acyl-CoA dehydrogenase
BB341_RS11520	-0.0850414	-1.1231355	0.258825	acyl-CoA dehydrogenase
BB341_RS26625	-4.5942068	0.36763736	-0.4893	Acyl-coa dehydrogenase protein
BB341_RS27680	-0.7439983	-1.286109	-0.04996	methoxymalonate biosynthesis protein
BB341_RS02890	-1.0929029	0.5493147	-1.31231	Putative enoyl coA hydratase
BB341_RS04840	0.60091801	-0.416236	1.135192	Enoyl-CoA hydratase
BB341_RS07030	1.70231939	1.45554699	0.126391	Putative enoyl-CoA hydratase
BB341_RS08735	-0.6107653	0.34929335	-0.05746	acyl CoA isomerase
BB341_RS11440	-0.8149848	-1.1723782	0.678472	enoyl-CoA hydratase
BB341_RS11705	-1.6807625	-1.6139403	0.367809	enoyl-CoA hydratase/isomerase
BB341_RS22000	-1.9833308	-0.6417635	0.091602	enoyl-CoA hydratase
BB341_RS22745	0.05047728	0.58168842	0.751403	Enoyl-CoA hydratase/isomerase
BB341_RS26945	-0.9377452	-0.584879	-0.06566	enoyl-CoA hydratase
BB341_RS28840	0.07011375	-1.7493497	0.155499	enoyl-CoA hydratase/isomerase
BB341_RS02945	0.23106395	1.03707597	-0.55487	Fatty acid oxidation complex alpha-subunit
BB341_RS04710	0.00055291	2.62868841	0.704797	Putative fatty acid oxidation complex alpha-subunit
BB341_RS11395	-0.4478853	-1.4606217	-0.04547	thiolase
**Dextrin converted into acetyl-CoA**				
BB341_RS20910	0.50574582	-0.4148002	0.36348	alpha-glucosidase
BB341_RS03865	0.2542761	-0.0705631	0.386314	Sugar kinase
BB341_RS21415	0.73002201	0.33100309	0.152367	Glucose kinase
BB341_RS22350	0.39088096	1.42631144	0.679817	Glucose-6-phosphate isomerase
BB341_RS07190	1.06622854	1.14323354	0.215213	6-phosphofructokinase
BB341_RS15270	0.68775958	2.4686042	0.766939	fructose-bisphosphate aldolase
BB341_RS22720	0.41871114	0.58214455	0.429531	aldolase
BB341_RS22335	-0.1495118	0.18410878	0.854916	bifunctional PGK/TIM

BB341_RS22325	-0.0470941	1.05223304	-0.13998	glyceraldehyde-3-phosphate dehydrogenase
BB341_RS22330	0.15702435	1.21710106	0.600905	phosophoglycerate kinase
BB341_RS23520	-0.1364709	0.78662715	0.484683	Phosphatase
BB341_RS07205	0.6928115	0.18140287	0.122512	pyruvate kinase
BB341_RS13605	1.13064441	0.47722467	0.771424	Branched-chain alpha-keto acid dehydrogenase E1-alpha subunit
BB341_RS13695	5.26085578	-0.9609545	1.817747	E1-alpha branched-chain alpha keto acid dehydrogenase
BB341_RS20205	-0.0730057	-0.0223998	0.866671	Pyruvate dehydrogenase subunit E1
BB341_RS21120	1.30061067	0.21044846	0.234593	2-oxoacid dehydrogenase subunit E1
**Key genes involved in glyoxylic acid cycle and gluconeogenesis**				
BB341_RS28800	0.953924274	7.660670041	0.111586634	Isocitrate lyase
BB341_RS28805	0.458194298	4.63162596	-0.372400971	Malate synthase
BB341_RS03955	0.744603343	1.790692171	1.769950243	Malate synthase
BB341_RS09375	-0.029674642	1.983425487	0.854895804	phosphoenolpyruvate carboxykinase
BB341_RS08985	1.228453773	2.037843087	0.995311174	fructose 1,6-bisphosphatase II
BB341_RS08395	0.967372956	0.183021401	-0.066289393	fructose-1,6-bisphosphatase
BB341_RS14335	0.563269082	0.572907237	-0.210144922	Sugar phosphatase
BB341_RS28280	-0.528115219	2.709903796	-0.797337808	Phosphoenolpyruvate synthase

Note: Red lettering indicates up-regulated genes. Green lettering indicates down-regulated genes.

Table S7 Comparison of transcriptional level of genes involved in Cephalomycin C biosynthesis in MH and ML medium.

genes	log2Fold Change ratio			Description
	MH24/ML24	MH48/ML48	MH96/ML96	
Cephalomycin C biosynthetic gene cluster				
pbpA	0.41124682	0.7230379	0.357661229	D-alanyl-D-alanine carboxypeptidase
cmcT	0.42201898	0.5119774	-0.896948022	cephamycin export protein cmcT
pcd	0.79674281	3.28407848	0.174628588	aldehyde dehydrogenase
cefE	0.40666228	4.7273551	-1.391786004	deacetoxycephalosporin C synthetase
cefD	1.10552362	3.32473237	-0.126410239	isopenicillin N epimerase
cmcI	0.86846349	5.21332074	-0.986208983	conserved hypothetical protein
cmcJ	1.06224628	4.47028723	-0.723629753	7-alpha-cephem-methoxylase P8 chain
cefF	0.34422829	3.91241886	-0.18456153	deacetoxycephalosporin C hydroxylase
cmcH	0.39845694	4.22598187	-0.292020343	hydroxymethylcephem-O-carbamoyltransferase
ccaR	0.61148169	1.7209061	1.392784745	positive regulator
blp	1.99717602	1.93551903	0.292908126	BLP
lat	0.76381374	3.74737123	-0.4179193	L-lysine-epsilon aminotransferase
pcbAB	0.98784806	3.86341425	-0.332430559	N-(5-amino-5-carboxypentanoyl)-L-cysteinyl-D-valine synthase
pcbC	0.37470567	3.26223795	0.196918101	isopenicillin N synthetase
pcbR	0.86795725	1.93770654	0.600818626	secreted penicillin binding protein
Genes involved in precursor metabolism of cephalomycin C				
BB341_RS00325	-0.22398199	-0.37985826	-0.197646564	asparagine synthetase
BB341_RS12885	0.13035015	-0.17718574	-0.383054405	hypothetical protein
BB341_RS15440	0.64232793	1.45553727	0.741991156	aspartokinase
BB341_RS15445	0.68521795	0.12933128	1.002032746	aspartate-semialdehyde dehydrogenase
BB341_RS19250	1.67084228	1.83835886	0.88345814	Aspartate-semialdehyde dehydrogenase
BB341_RS18780	-2.55234477	-1.51667894	-0.366256707	DUF993 domain-containing protein
BB341_RS06710	-0.354789	4.16141035	0.214968799	acetohydroxy acid synthase small subunit
BB341_RS06715	-0.11419988	1.90396877	-0.009007091	Acetolactate synthase
BB341_RS07365	0.61788151	0.62589272	1.091984751	3-hydroxyacyl-CoA dehydrogenase

Note: Red lettering indicates up-regulated genes. Green lettering indicates down-regulated genes.

Table S8 Primers used in this study.

Oligonucleotides	DNA Sequence (5'→3')
16S-RT For	GAGATCCGCCTTCGCCACCG
16S-RT Rev	CTGCATTCGATACGGGCAGGC
ceaS2-RT For	AGGCCGCGTCGATTCTCTTCG
ceaS2-RT Rev	AGAGGTTGGTCATACCGGGGC
claR-RT For	TGCTGTCGCTGGTCTCCACG
claR-RT Rev	TAGGCCGCGTCCACCTGGTA
cad-RT For	CCGACTGGACCCGGATGATCG
cad-RT Rev	TTCGTGGCCTGGTAGACGGC
gcas-RT For	CACCCCTGGCCGACTATGCC
gcas-RT Rev	GCCCGTGGGTGTACCAGGAC
argC-RT For	CCTGCGAGAACGCCGTCAGC
argC-RT Rev	GACGGTACGGGTGGCGGTTG
argJ-RT For	TGGCGTGGGTGTCCTGGAAG
argJ-RT Rev	ATCTGGCACTGGTGGTGAAC
argB-RT For	AGATTGAGCAGCCCCACCAG
argB-RT Rev	ACATCGTCTTTCTCCACCAC
argH-RT For	ATGTCGGGGTTCTTCTTCTG
argH-RT Rev	AGTTCGCCTTCATCACCGCC
bls2-RT For	TGCCGCTGTACACCTGTGTGG
bls2-RT Rev	CGCGGGCACCTGGTAGACAC
pah2-RT For	ACGGCGCAGAGCCATCTGTC
pah2-RT Rev	TTGGTGTCGGAGTGCGCGTC
cas2-RT For	CTCCGAGCTTCCCGAGGTGC
cas2-RT Rev	CGCGCAGCAGCAGATAACCG
bls1-RT For	GAACTCGTCAGGGAACTGCC
bls1-RT Rev	GGACGGGGGACATCTCGTTG
cyp450-RT For	AGCCAGGTGTGGCTGGTGAC

cyp450-RT Rev	GCGGATGAACGACGCCGACT
fd-RT For	GCCCCCGAGATCTTCGACCAG
fd-RT Rev	TAGCCCTCGGTGACCGTGAT
pah1-RT For	GCAGGAGCGGTCTCGGAGG
pah1-RT Rev	GCTCCCGTTCCCGTACCGAC

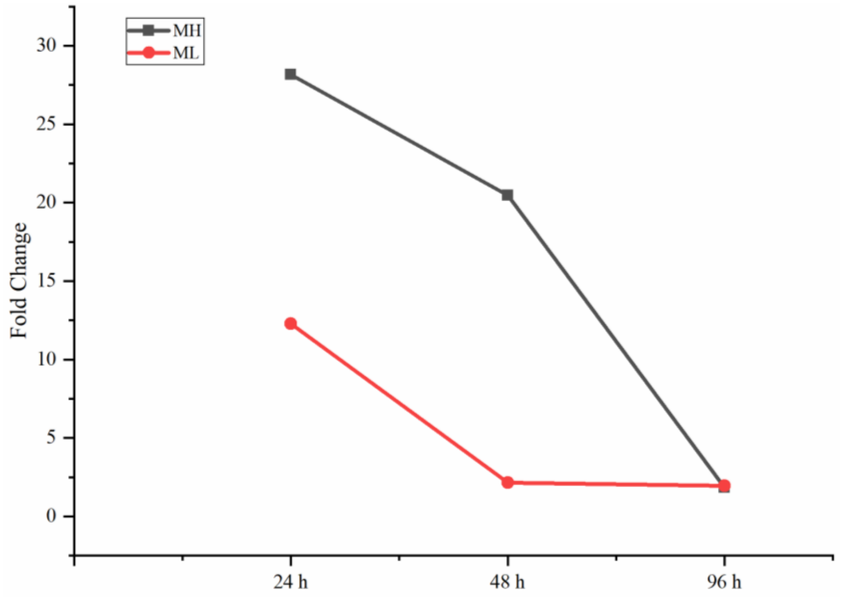

Fig. S1 Transcriptional trend analysis of CA biosynthesis gene clusters in MH and ML medium

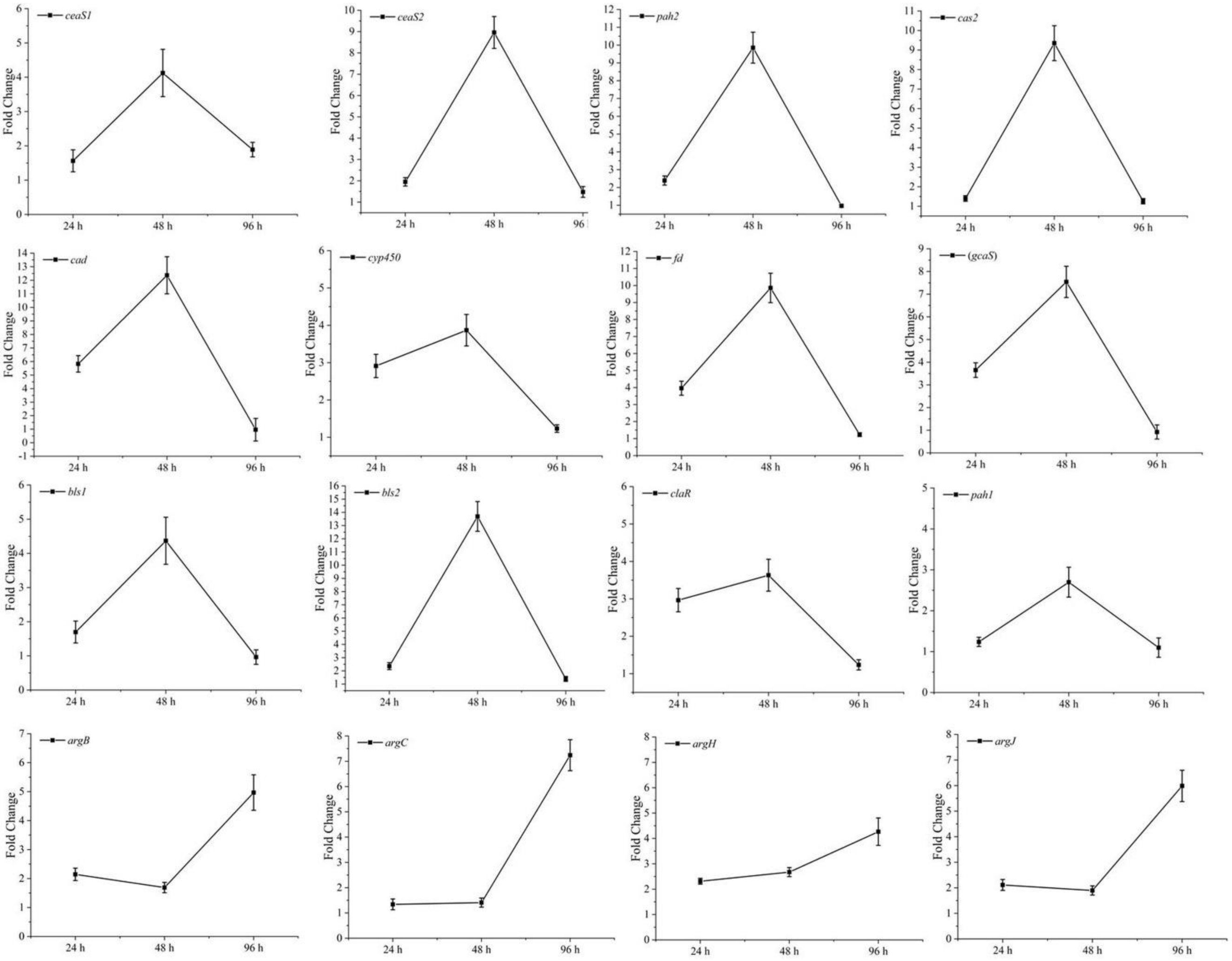

Fig. S2 Expression of key genes involved in CA and arginine biosynthesis by RT-qPCR in *Streptomyces clavuligerus* F613-1. Results were normalized for 16S rDNA gene content and are shown as fold change over the ML medium control, which was given a value of 1. Fold change: expression level in MH medium compared with ML medium. Data are the mean ± SD of three separate experiments.
